# Lithium-Induced Brugada Pattern: A Case Report and Review of Literature

**DOI:** 10.7759/cureus.9351

**Published:** 2020-07-23

**Authors:** Venkatesh Ravi, Nicholas J Serafini, Priyanjali Pulipati, Richard Trohman, Parikshit S Sharma

**Affiliations:** 1 Cardiology, John H Stroger, Jr. Hospital of Cook County, Chicago, USA; 2 Division of Cardiac Electrophysiology, Rush University Medical Center, Chicago, USA; 3 Internal Medicine, St. Joseph Mercy Oakland Hospital, Pontiac, USA; 4 Electrophysiology, Rush University Medical Center, Chicago, USA

**Keywords:** lithium, brugada pattern, sudden cardiac death

## Abstract

Lithium-induced type 1 Brugada pattern in asymptomatic patients is an uncommon occurrence that is challenging to manage and to estimate the risk of sudden cardiac death (SCD). We describe a case of a 74-year-old woman who presented with type 1 Brugada pattern while on lithium therapy. Her lithium level was within the therapeutic range at the time of presentation. There was no evidence of ventricular ectopy or malignant arrhythmias. Review of electrocardiogram (ECG) prior to initiation of lithium therapy demonstrated type 3 Brugada pattern. Lithium was promptly discontinued, and the patient was closely monitored in the hospital for 48 hours with serial ECGs and telemetry, as her lithium levels decreased. The Brugada pattern resolved on day 10 of discontinuation of lithium therapy and no further intervention was performed. Early diagnosis and prompt discontinuation of lithium leads to the resolution of type 1 Brugada pattern and may reduce the risk of SCD. The case highlights the importance of obtaining baseline ECG when initiating lithium especially in patients with type 2 or 3 Brugada pattern and provides an overview of the serial changes in ECG pattern until resolution following discontinuation of lithium. Electrophysiology study for risk stratification in asymptomatic patients does not appear to provide any additional benefit.

## Introduction

Brugada syndrome is an inherited disorder associated with the risk of ventricular fibrillation and sudden cardiac death (SCD) in a structurally normal heart. The prevalence of Brugada pattern in the general population ranges from 0.1% to 1%, and it is estimated to account for up to 4% of all SCD in the general population [1,2]. Unmasking of type 1 Brugada pattern by class 1 antiarrhythmics (sodium channel blockers) has been shown to be helpful in risk stratification of patients with syncope or family history of SCD [3,4]. Lithium is a drug widely used as a mood stabilizer with limited evidence on its capacity to block sodium channels. In mice transfected with the SCN5A gene (known to be commonly involved in Brugada syndrome), lithium selectively blocked cardiac sodium channels, even at therapeutic levels [5]. There are a few reported cases of lithium-induced Brugada pattern on ECG in humans as well. However, there is a lack of clarity on optimal management and estimating the short-term risk of malignant arrhythmias or the long-term risk of SCD in asymptomatic patients [6]. Other unanswered aspects include the duration of ECG changes, duration of monitoring required, whether there is a linear dose-response relationship to the pattern, and the role of electrophysiology study (EPS) in risk stratification. We describe a case of an asymptomatic patient with type 1 Brugada pattern unmasked by lithium.

## Case presentation

**Figure 1:**
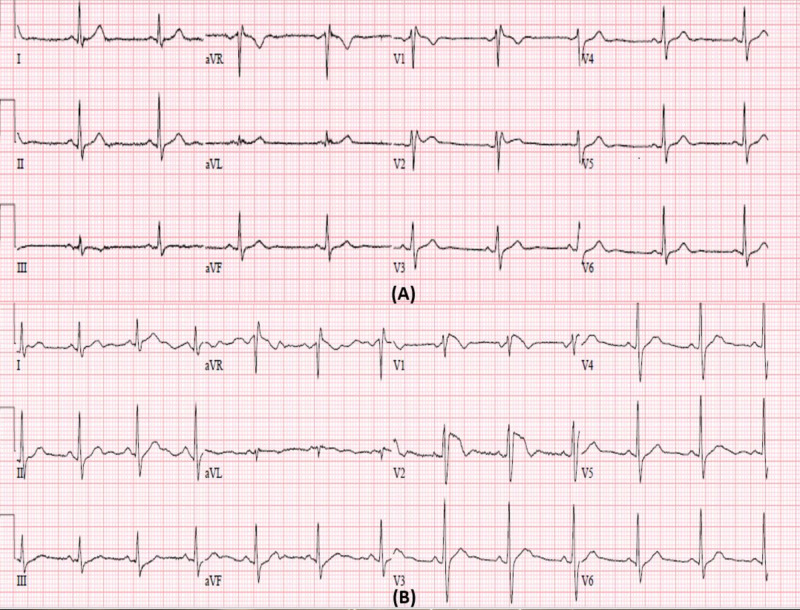
(A) Baseline electrocardiogram (ECG) at the time of initiation of lithium showing sinus rhythm with evidence of saddleback ST elevations <2 mm suggestive of type 3 Brugada pattern in V2. (B) ECG on presentation, nine days after initiation of lithium showing sinus rhythm with coved ST elevation >2 mm in V1 and V2.

A 74-year-old woman was admitted to the geriatric-psychiatry unit with an extended acute episode of mania with persecutory paranoia. While undergoing titration of multiple psychiatric medications, the cardiology service was consulted for an electrocardiogram (ECG) for QT monitoring. The ECG was found to be abnormal with ST elevations in leads V1 and V2. On initial evaluation, she was asymptomatic, denying chest pain, dyspnea, dizziness, palpitations, or syncope. The patient’s past medical history was significant for benign frontal meningioma and bipolar disorder. There was no family history of SCD among first-degree relatives as well as among extended family members. She had been initiated on olanzapine 5 mg twice daily and lithium 450 mg twice daily eight days prior to presentation. Her vitals on presentation were within normal range, and physical exam was normal. The baseline ECG prior to initiation of lithium therapy demonstrated sinus rhythm with <2 mm saddleback pattern ST elevations in V1 and V2 suggestive of type 3 Brugada pattern (Figure 1A) and QTc interval of 400 ms. The ECG at the time of presentation revealed normal sinus rhythm with coved ST elevations >2 mm in V1 and V2 consistent with type 1 Brugada pattern (Figure 1B) and QTc interval of 420 ms.

**Figure 2:**
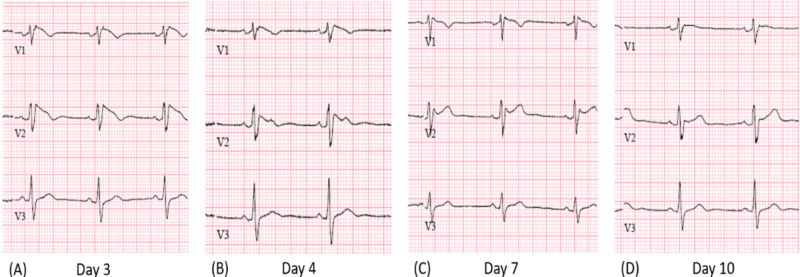
Serial electrocardiograms, labeled in order of time in days from the discontinuation of lithium. (A) Persistence of type 1 Brugada pattern. (B) Type 2 Brugada pattern. (C) Type 2 Brugada pattern. (D) Type 3 Brugada pattern.

The initial routine laboratory workup was unremarkable. Her lithium level was 1.01 mmol/L (reference range 0.4-1.3 mmol/L). She was diagnosed with the Brugada pattern unmasked by lithium and admitted to inpatient service. Lithium was discontinued, and antipsychotics known to prolong action potential duration were held. She was monitored on telemetry in the hospital for the duration of the half-life of lithium. There was no evidence of ventricular ectopy or arrhythmias captured on telemetric monitoring. Serial ECG monitoring was documented (Figure 2) as her lithium levels decreased. She was transferred back to the geriatric-psychiatry unit after 48 hours of inpatient monitoring where daily ECGs were continually followed. By day 4, the patient’s ECG demonstrated resolution of the type 1 Brugada pattern but began manifesting the type 2 Brugada pattern (Figure 2B). On day 10, there was a resolution of the type 2 Brugada pattern, and her ECG returned to her baseline (Figure 2D). No additional intervention was performed, and the patient continued to do well during follow-up.

## Discussion

We described a patient who had lithium unmasked type 1 Brugada pattern which was incidentally noted when she presented with an episode of acute mania and resolved by day 10, following the discontinuation of lithium. The case highlights the importance of obtaining baseline ECG when initiating lithium especially in patients with type 2 or 3 Brugada pattern and provides an overview of the serial changes in ECG pattern until resolution following discontinuation of lithium. 

Lithium has been known to have potentially cardiotoxic effects for several decades; however, the first two cases of lithium unmasked Brugada syndrome were reported in 2005 [5]. There were 13 reported cases of lithium unmasked Brugada pattern on ECG as shown in Table 1 [5,7-16]. Including our patient, ten were male and four were female. Their presenting complaints were as follows: five (36%) with abnormal ECG, four (29%) with psychiatric symptoms, two (14%) with syncope, one (7%) with cardiac arrest, one (7%) with palpitations, and one (7%) with septic shock. Among six patients with documented baseline ECG, two (33%) had type 3 Brugada pattern at baseline. Three (27%) patients exhibited supratherapeutic lithium values among the 11 patients with documented lithium values. The dose of lithium ranged from 300 mg daily (N = 1) to 900 mg daily (N = 5) among the eight patients with documented daily dosing of lithium. Lithium was used in combination with at least one additional antipsychotic medication in six patients. Lithium was discontinued on presentation in 13 out of the 14 patients, while in one patient, lithium was continued due to severe bipolar disorder. The average duration of persistence of ECG pattern was 21 days, with the earliest resolution at five days from discontinuation and the longest persistence at 49 days. Among the total cases, five (36%) patients had either a history of syncope or SCD consistent with the diagnosis of Brugada syndrome, five (36%) patients had no prior history of syncope or SCD suggestive of isolated unmasking of the Brugada pattern on ECG, and four (29%) patients had insufficient documented information. Among the five patients with diagnosed Brugada syndrome, all had implantable cardioverter-defibrillator (ICD) implanted. Among the five patients with asymptomatic Brugada pattern, none had inpatient EPS or ICD placement, but one of them was planned for an outpatient ICD. Among the four patients with insufficient information, none had an EPS or ICD placement. There was only one reported death in a patient who was planned for outpatient evaluation with an EPS. The cause of death was unclear but was reported as sudden death [8].

**Table 1:** Demographic and clinical characteristics of the 13 previously reported patients with lithium-induced type 1 Brugada pattern. N: serial number; M: male; F: female; ECG: electrocardiogram; SCD: sudden cardiac death; SHD: structural heart disease; TTE: transthoracic echocardiogram; EPS: electrophysiology study; AICD: automated implantable cardioverter-defibrillator; SCN5A: sodium voltage-gated channel alpha subunit 5; AS: aortic stenosis; WPW: Wolff-Parkinson-White; TCA: tricyclic antidepressant; DC: discontinued; NA: not available; OP: outpatient; f/u: follow-up; NSVT: non-sustained ventricular tachycardia; VT: ventricular tachycardia; VF: ventricular fibrillation.

N	Author/year	Age/sex/prior cardiac history	Chief complaint	Presenting ECG	Prior symptoms/family history of SCD	Psychiatric medicine dose/duration/drug level (therapeutic range)	Baseline ECG	Duration of type 1 Brugada pattern ECG persistence/repeat ECG	Psychiatric medication change	Death reported	TTE or cardiac MRI/any SHD	EPS for inducible VT/VF	AICD	SCN5A gene
1	Darbar et al. [5]/2005	26/M/none	Abnormal ECG	Type 1	‘Fading out’ since teen/none	Lithium 900 mg daily/2 months/1 meq/L (0.8-1.2)	Normal	6 weeks/type 2 pattern, ST elevation > 2 mm	Lithium DC. Lamotrigine + clonazepam	No	MRI/no SHD	Yes, inducible VF	Yes	No
		65/M/none	Syncope	Type 1	Recurrent syncope/yes	Lithium/20 years/NA	NA	NA/type 3 pattern	Lithium reduced to 400 mg daily	No	TTE/mild AS	Yes, inducible NSVT	Yes	No
2	Strohmer et al. [7]/2006	64/F/none	Abnormal ECG	Type 1	Recurrent syncope/NA	Lithium 300 mg daily/4 weeks/0.2 mmol/L (0.5-1)	Normal	1 week/type 3	Lithium DC	No	TTE/no SHD	Yes, inducible VF	Yes	NA
3	Roberts-Thomson et al. [8]/2007	39/M/none	Acute mania	Type 1, QT 540 ms	NA/NA	Lithium 200 BID, valproate 500 mg BID, amitriptyline 200 mg daily, haloperidol 10 mg TID)/NA/0.2 mmol/L (0.5-1)	NA	NA/normalized	All psych meds DC	Yes. Sudden death before f/u	NA	NA	No	NA
4	Fragakis et al. [9]/2007	58/F/WPW (EPS)	Abnormal ECG	Type 1	None/none	Lithium 900 mg daily, propafenone/NA/0.8 meq/L	Pre-excitation, type 3	NA	Lithium continued. Propafenone DC	No	NA	No	No	NA
5	Laske et al. [10]/2007	42/M/none	Syncope	Type 2	Recurrent syncope/none	Lithium/8 years/0.75 mmol/L (0.5-1)	NA	7 weeks/normal	Lithium DC. Started olanzapine	No	NA	Yes, inducible NSVT	Yes	NA
6	Pirotte et al. [11]/2007	42/M/none	Abnormal ECG	Type 1	None/NA	Lithium 300 mg TID/5 months/1.4 meq/L (0.8-1.4)	Normal	5 days/type 2 pattern	Lithium tapered and DC	No	NA	NA	No. OP plan	NA
7	Chandra et al. [12]/2009	38/M/none	Cardiac arrest	Type 1	Cardiac arrest/NA	Lithium 900 mg daily, lamotrigine, ziprasidone)/1 year/0.7 meq/L	NA	NA	Lithium DC	No	TTE/no SHD	No	Yes	NA
8	Wright et al. [13]/2010	49/M/none	Ataxia, tremors	Type 1	None/none	Lithium, carbamazepine, risperidone)/NA/2.5 mmol/L (0.8-1.2)	NA	NA/type 1 at lithium level 1.9 mmol/L. Normal at 0.8 mmol/L	Lithium DC	No	NA	No	No. OP f/u plan	NA
9	Kofune et al. [14]/2013	60/M/none	Depression	Type 1	NA	Lithium 400 mg/day, clomipramine, nortryptiline/NA/NA	Normal	21 days/normal	Lithium, TCA DC	No	NA	No	No	NA
10	Crawford et al. [15]/2015	58/M/none	Unresponsive	Type 1	NA	Lithium/NA/2.4 mmol/L (0.8-1.2)	NA	11 days/normal	Lithium DC	No	TTE/no SHD	No	No	NA
11	Gentille et al. [2]/2015	50/M/none	Septic shock	Type 1	None/none	Lithium, clozapine, citalopram/NA/1.89 meq/L (1-1.2)	NA	NA normal at lithium level 0.32 meq/L	Lithium DC	No	TTE/no SHD	No	No	NA
12	Asil et al. [16]/2017	48/F/none	Palpitations	Type 1	None/none	Lithium/NA/0.91 mmol/L (0.6-1.2)	NA	7 days/normal	Lithium DC	No	NA	No	No	NA

Patients who have unmasking of Brugada pattern with lithium may have a higher risk of long-term SCD. This is based on the known information of the increased risk of SCD with Vaughan-Williams class I agents or sodium channel blockers when unmasking the Brugada ECG pattern [3,4]. Normalization of the ECG pattern may lead to an underestimation of the prevalence of the disease placing some patients at increased risk [3]. Among Brugada syndrome patients who survived ventricular fibrillation, those with transient Brugada pattern unmasked by ajmaline, procainamide, or flecainide had a similar risk of recurring arrhythmic events (ventricular fibrillation or sudden death), compared with patients with persistent Brugada pattern. When patients with symptomatic and incidental type 2 or type 3 Brugada pattern underwent provocation with ajmaline or flecainide, those who had unmasking of type 1 pattern had a higher risk of sudden death, syncope, and appropriate ICD intervention (p = 0.01) [4]. However, in the subgroup analysis of asymptomatic individuals with a type 2 or type 3 pattern, the incidence of events was low, regardless of the sodium channel blocker test result (p = 0.18). Although patients who had history consistent with Brugada syndrome and unmasking of type 1 Brugada pattern on ECG by lithium have a higher risk of SCD, asymptomatic patients with lithium-induced type 1 Brugada pattern may not be at an increased risk of SCD. Hence, it is crucial to obtain a comprehensive family history and evaluate prior cardiac symptoms in these patients. Patients initiated on lithium might benefit from routine pre-initiation ECG and at least one follow-up ECG, especially in those with type 2 or type 3 Brugada pattern at baseline.

Patients may also be at increased short-term risk of acute malignant arrhythmias for the duration of persistence of type 1 Brugada pattern with coved ST-segment elevation. The increased cardiovascular mortality rate in patients with bipolar disorders may be attributed to the proarrhythmic effects of psychotropic drugs in patients with underlying cardiac disease [17-19]. In our review of literature, there were no reported malignant arrhythmias during the time of persistence of type 1 Brugada pattern. However, among the 14 patients, there was one (7%) reported sudden death of unclear etiology following discharge and prior to a follow-up appointment. Based on prior evidence, this one case of sudden death of unclear etiology may not suggest that asymptomatic patients with lithium unmasked type 1 Brugada pattern are at high risk of short-term cardiac events. However, further studies are required to answer this question. Inpatient monitoring for about 36-48 hours, which corresponds to the initial half-life of lithium or until lithium levels are therapeutic, appears to be reasonable [5,7-16]. Monitoring as an inpatient until resolution of the Brugada pattern may not offer any additional benefit and would lead to increased cost burden. 

Risk stratification in patients with lithium unmasked Brugada pattern on ECG is challenging. Initially, variables recommended for risk stratification were history of syncope, family history of SCD, or EPS inducibility [5]. However, the utility of induction of malignant arrhythmias by EPS in risk stratification of asymptomatic patients with spontaneous or drug-induced Brugada pattern is controversial. As an independent predictor of risk of malignant arrhythmias in asymptomatic patients with type 1 Brugada pattern, EPS had a hazard ratio (HR) of 0.721 (CI 0.2-2.2, p = 0.559), sensitivity of 36%, a specificity of 59%, a positive predictive value of 3.9%, and a negative predictive value of 95% [20]. Hence, EPS is not recommended for risk stratification. More reliable independent predictors of the risk of malignant arrhythmias were found to be syncope and spontaneous type 1 Brugada pattern on ECG which had an HR of 6.4 (CI 2.2-18.6, p = 0.001); QRS fragmentation which had an HR of 8.9 (CI 3.0-26.0, p < 0.001); and ventricular refractory period <200 ms which had an HR of 5.7 (CI 1.6-20.3, p = 0.008). QRS fragmentation was defined as two or more spikes within the QRS complex in V1-V3.

## Conclusions

In asymptomatic patients with type 1 Brugada pattern unmasked by lithium, prompt discontinuation of lithium and hospital observation for 48 hours or until lithium levels are in or below therapeutic range appears to be reasonable. Asymptomatic patients with lithium-induced type 1 Brugada pattern may not have a higher risk of SCD. Patients initiated on lithium may benefit from routine pre-initiation ECG and at least one follow-up ECG in those with type 2 or type 3 Brugada pattern at baseline. EPS does not appear to provide additional benefit in the risk stratification of asymptomatic patients and is not routinely recommended.
